# Supplementary material for: Towards implementation of context-specific integrated district mental healthcare plans: A situation analysis of mental health services in five districts in Ghana
Source: PLoS One. 2023 May 18;18(5):e0285324. doi: 10.1371/journal.pone.0285324 (PMC10194950; doi:10.1371/journal.pone.0285324)
Supplement: Attachment — Submitted filename: COMMENTS & SUGGESTION.docx [file pone.0285324.s001.docx]

PLOS ONE

**Manuscript Title**- Towards implementation of context-specific integrated district mental health care plans: A situation analysis of mental health services in five districts in Ghana.

**Manuscript Number**: PONE-D-22-23817

This manuscript is not written in Standard English and is not presented in an understandable manner overall. So, it needs to be revised and rewritten in a concise and clear manner.

As a limitation of this situational analysis, focused group discussions were not held with communities and beneficiaries of mental health services. This was a missed opportunity to explore their feelings and experiences in-depth regarding mental health disorders and the treatment options available in health facilities and the community.

**Abstract:** Despite having all scientific material, it is poorly written and needs to be thoroughly rewritten.

**Background:** It lacks information on other countries' trends regarding the integration of mental health services into non-specialized health facilities. It is better if other countries' trends are incorporated into the background section of this study.

Lines 131–133: The primary objective of this situational analysis is not in line with the aim stated in the abstract.

**Methods**: Line 138, "Methods, can be correct as "Methods and materials."

Line 140, "Design," and Line 143, "Setting," can be corrected as "Study setting and design." And it needs a summary in one paragraph.

In lines 143–145, you stated that the study sites were selected by key mental health stakeholders. That means the study sites were chosen on purpose by stakeholders; do you believe the districts chosen on purpose represent other Ghana districts?

The study period for this situational analysis is not clearly described within the section on "Methods and Materials."

Line 173-participants can be corrected as "secondary data source" and "study participants."

It is not clear how secondary data were collected, cleaned, and transferred to analysis.

Lines 177–179: insert a separated subtopic as "Ethical considerations."

**And the rights of study participants were not clearly and specifically stated as ethical considerations.**

Line 181, "Measures," can be corrected as "Data collection tools and procedures."

It is better to include "Data quality management" as separate sub-headings.

Line 202, "Data analysis," can be corrected to "Data processing and analysis."

**Results**: - Generally, the result section is too long and broad to read and easily understand within a short period of time. So, it needs to be summarized in a clear and brief manner.

Lines 214–222, you stated the source of data for this situational analysis. It is better if sources of data are only stated in the Methods and Materials section rather than in the Results section.

Line 225, "Socio-demographic and economic context," can be corrected as "Socio-Demographic and Socioeconomic Characteristics."

Line 246, "health profile," instead rewrite it as "health structure" or "health tire system."

Did Ghana have a mental health treatment guideline?

Did all health institutions located in the study district have mental health treatment guidelines?

Line 271, "mental health services organization," should instead be rewritten as "mental health police and regulation."

Lines 273-282 tell the story of Ghana's mental health authorities. Mental health authorities and offices are not mental health organizations, and their main responsibilities might be recruiting mental health professionals, planning mental health services, coordinating, planning, and conducting supportive supervision, delivering training for health providers, and conducting monitoring and evaluation. Since the Mental Health Act is not part of the study results, it would be better if Ghana’s Mental Health Act 2012 (Act 846) were incorporated into the background of this section of the study.

Lines 284–297 described mental health services plans, authorities, responsibilities, and roles at the regional and district level. What about mental health care plans at each and every level of mental health facilities (i.e., at health posts, health centers, district hospitals, and tertiary hospitals)? Similarly, this situational analysis did not report results showing health facilities' quarterly and annual plans or performances regarding mental health services at health facilities.

It is better if you summarize the two subtopics (availability of mental health services and human resources for mental health service provision) as follows: mental health professionals and mental health services at health facilities

You stated that people with mild and moderate mental and neurological disorders got mental health services at community health centers and district hospitals. What about people with severe mental illnesses who met all admission criteria and require close monitoring?

Line 384, table 3, description, needs revision. Please revise and rewrite it in a clear and brief manner.

The topic of the first column of table 3 was not appropriately written; revise it as "mental health activities at mental health offices and health institutions."

In Table 3 column 1, topic 2, you state "personnel type." Rephrase it as "mental health professionals from various backgrounds."

According to this situational analysis, alcohol detoxification was possible without institutionalization in Tolon, Asunafo North, and Anloga districts. How is alcohol detoxification possible without admitting the clients to health facilities? What are the researcher’s justifications?

You have reported that severe mental health illness is treated at home with the support of families in cases from all study districts. But it is difficult to treat SMI at the home level. According to the mhGAP intervention guidelines, all individuals with SMI should be treated by a psychiatrist at a psychiatry clinic or unit.So, what are your justifications for this inappropriate treatment service for people with SMI?

What are the parameters of mental health service coverage? What are the incidence and prevalence of mental, neurological, and substance use conditions in your study districts? In order to measure or estimate mental health services and coverage at study sites, incidences and prevalence of mental health disorders must be stated.Your situational analysis did not show the actual number of people with MNS conditions who had gotten treatment at health facilities in study districts. Furthermore, these situational analyses fail to provide a clear and concise description of the treatment gaps in the study area.

Lines 364-366 require revision because they are unclear and the write-up is inadequate.

Line 374-375, table 4, 19 words, which is more than the standards, needs revision.

Lines 384-385, able-5, 22 words, which exceeds the plos one journal standards

Line 387, subtopic "Availability and sources of psychotropic medication."

Similarly, the availability of a mental health plan, budget allocation, coordination, integration, and use of screening tools are not appropriately stated for each district's health offices.

Revise the paragraph as "psychotropic medication for people with MNS conditions" and try to summarize and rewrite the availability and constant supply of essential psychotropic drugs by kinds (i.e., anti-psychotics, antihistamines, anti-depressants, and anxiolytics) at each and every level of health facilities.

Table 6, last row and column 6, "Carbamazepine," is available at Asunafo North for epilepsy but not for mood stabilizers. Please, would you like to justify the reason behind these discrepancies?

Line 419, under subtopic "monitoring and evaluations," Did the district health offices and health facilities have clear plans to conduct monitoring and evaluation programs at each level of health facility? Did they have a well-established functional monitoring and evaluation team?

This paragraph under the subtopic "monitoring and evaluation" is not clear and concise enough to be read and understood. So, please summarize and rewrite this paragraph in a clear and brief manner.

Discussion: - lacks depth, is too shallow, and does not address other studies' findings from around the world. It is better to include "study limitations and strengths" as separate sub-headings.
