# Supplementary material for: Towards implementation of context-specific integrated district mental healthcare plans: A situation analysis of mental health services in five districts in Ghana
Source: PLoS One. 2023 May 18;18(5):e0285324. doi: 10.1371/journal.pone.0285324 (PMC10194950; doi:10.1371/journal.pone.0285324)
Supplement: Attachment — Submitted filename: Response to reviewers_PONE-D-22-23817.docx [file pone.0285324.s002.docx]

Dear Prof Chenette,

Manuscript: PONE-D-22-23817, Towards implementation of context-specific integrated district mental health care plans: A situation analysis of mental health services in five districts in Ghana

Thank you for your comments regarding our manuscript and the invitation to revise and resubmit. Please see below a table summarising the response to the concerns raised by the reviewers. All the revisions in the revised manuscript are in track changes or highlighted.

| **Comment/concern** | **Response/action** |
| --- | --- |
| Reviewer 1 |  |
| 1. English editing will be of great value to this paper. There are long sentences. The background of the paper is not having a clear paragraph direction and therefore makes it difficult to determine the direction of the study. Some terms should be defined in the first use. E.g “the PRIME” study. The study's main aim is not well stated in the manuscript | Thank you for your comment. We have now done some further editing (to improve our English language score of 80% + when we submitted the preprint) and shortened long sentences whilst maintaining meaning. The background in particular has been tightened a bit with clear paragraphs to make the flow better. Essentially, we sought to make the case for improving access to mental health services as a key strategy to narrowing the treatment gap. One way to achieve this is through context-informed mental healthcare plans. Before these are deployed however, we propose the utility of situation analysis as an approach to carefully understand the healthcare system and identify opportunities.  We have also now restated the study aim in the background to ensure consistency with the abstract. |
| 2. The paper does not really follow a proper methodology reporting. This makes it difficult to gather information. It would be helpful to have sections where the study setting, study design, sampling procedures, data collection procedures and data collection instruments, inclusion and exclusion criteria, data analysis, etc. are clearly described. The current methodological draft is a mishmash of all of these sections in various combination. | Thank you for your comment. We have now ensured a more thorough description of the methods applied in this situation analysis. |
| 3. Result- The type of data collected and the data collection procedures should be described in the methods section. | Thank you. This is well noted and has been effected. |
| 4. The discussion should be tightened up significantly - There are some text or statements without references. A concrete storyline is not visible. The discussion should be more succinct, with more literature supporting the argument/perspective the authors would like the reader to appreciate. It should clearly describe the social and policy implications of the study findings. | Thank you for your comment. We have now worked a bit more on the discussion in terms of improving the flow but also including appropriate references. Our story as discussed was to reiterate the huge treatment gap and poor mental health infrastructure in Ghana, and how this story is similar to other LMIC settings reported in the PRIME studies. Beyond this, we sought to highlight the many implications of our findings in terms of policy and legislation, health systems for mental health, and opportunities for improving mental health services.  We concluded with thoughts on how to improve access through the innovative use of district mental healthcare plans and how this situation analysis has provided critical data to support the implementation of such an intervention. |
| Reviewer 2 |  |
| 1. Abstract: Despite having all scientific material, it is poorly written and needs to be thoroughly rewritten. | Thank you for your comment. We have now worked a bit more on the abstract. |
| 2. Background: It lacks information on other countries' trends regarding the integration of mental health services into non-specialized health facilities. It is better if other countries' trends are incorporated into the background section of this study. | Thank you for your comment.  The background has been tightened a bit with clear paragraphs to make the flow better. Essentially, we sought to make the case for improving access to mental health services as a key strategy to narrowing the treatment gap. One way to achieve this is through context-informed mental healthcare plans. Before these are deployed however, we propose the utility of situation analysis as an approach to carefully understand the healthcare system and identify opportunities.  As mentioned in the background, we recognise the challenges with integrating mental healthcare in primary health following Cohen’s research. We also do now know that the Programme for Improving Mental health carE (PRIME) has generated a substantial volume of evidence on how to tackle some of these challenges, but also importantly, the impact on patient outcomes. The PRIME programme was conducted across five LMICs (Ethiopia, India, Nepal, South Africa and Uganda. |
| 3. Lines 131–133: The primary objective of this situational analysis is not in line with the aim stated in the abstract. | Thank you. We have now restated the study aim in the background to ensure consistency with the abstract. |
| 4. Methods: Line 138, "Methods, can be correct as "Methods and materials." | Thank you. We have now made this suggested change that’s also in line with the Journal’s manuscript structure. |
| 5. Line 140, "Design," and Line 143, "Setting," can be corrected as "Study setting and design." And it needs a summary in one paragraph. | Thank you for this suggestion. We have now merged some of these sub-headings but respectfully request to keep ‘study design’ separately. |
| 6. In lines 143–145, you stated that the study sites were selected by key mental health stakeholders. That means the study sites were chosen on purpose by stakeholders; do you believe the districts chosen on purpose represent other Ghana districts? | Thank you for this great question. The selection of the study districts was guided by a set of criteria borrowed from the PRIME studies and roundly endorsed by the key stakeholders. The criteria included: Geo-political equity (each of the five districts with health facilities should spread across the three zones of Ghana: Southern, Middle Belt, and Savannah); representativeness (i.e. the districts should not be over-resourced, but also not under-resourced, particularly in relation to human resources so that lessons could be generalised to inform scale up in other districts); an appropriate level of prevailing mental health activity (i.e. there should be no existing sites for mental health research or previous/ or on-going district mental health plans or national academic centres for mental health should exist as this would create an unrealistic environment which is not representative of other districts; and willingness of district leadership to engage.  Each of these districts will thus be representative of one or more districts in Ghana. |
| 7. The study period for this situational analysis is not clearly described within the section on "Methods and Materials." | Thank you for this observation. We have now provided this important detail in the methods. |
| 8. Line 173-participants can be corrected as "secondary data source" and "study participants." | Thank you for this useful suggestion. We have now provided this separate sub-heading. |
| 9. It is not clear how secondary data were collected, cleaned, and transferred to analysis. | Thank you for drawing our attention to this. Secondary data were collected using relevant sections of the PRIME situation analysis tool. As reported in our results the sources included: annual health reports of each district covering 2019 and 2020; mental health service delivery statistics reported in the District Health Information Management System (DHIMS) for 2020; medium-term development plans of the districts; the 2010 Population and Housing Census (Ghana has a more recent 2020 census but this was not available at the time of data collection for this study); and District Analytical Reports. Data were recorded directly on the relevant sections of the tool, checked for accuracy, and thereafter entered on an excel sheet for data management and analysis. |
| 10. Lines 177–179: insert a separated subtopic as "Ethical considerations." 11. And the rights of study participants were not clearly and specifically stated as ethical considerations. | Thank you for this suggestion. We have now provided a separate sub-heading for ethical considerations. |
| 12. Line 181, "Measures," can be corrected as "Data collection tools and procedures." | Thank you for this suggestion. We have now renamed the sub-heading accordingly. |
| 13. It is better to include "Data quality management" as separate sub-headings. | Thank you for this suggestion. We have now included a separate sub-heading on data quality management. |
| 14. Line 202, "Data analysis," can be corrected to "Data processing and analysis." | Thank you for this suggestion. We have now renamed the sub-heading accordingly. |
| 15. Results: - Generally, the result section is too long and broad to read and easily understand within a short period of time. So, it needs to be summarized in a clear and brief manner. | Thank you for your comment. We reckon this is because of the nature of the study and the data. Some discussion within the results is almost inevitable for a situation analysis report. Nonetheless, we have tried to cut-back on some of the text. |
| 16. Lines 214–222, you stated the source of data for this situational analysis. It is better if sources of data are only stated in the Methods and Materials section rather than in the Results section. | Thank you for the suggestion. We had information on data sources as part of results because we felt these form part of the results in terms of what was eventually used for the analysis (compared to what we’d planned to do).  We have however taken your suggestion and moved this information to the methods section. |
| 17. Line 225, "Socio-demographic and economic context," can be corrected as "Socio-Demographic and Socioeconomic Characteristics." | Thank you for this suggestion. We have now renamed the sub-heading accordingly |
| 18. Line 246, "health profile," instead rewrite it as "health structure" or "health tire system." | Thank you for this suggestion. We have now renamed the sub-heading as ‘General and PHC context for integrating mental health care’ |
| 19. Did Ghana have a mental health treatment guideline? | Our study could not identify any standard treatment guidelines, particularly for psychological treatments. |
| 20. Did all health institutions located in the study district have mental health treatment guidelines? | Our study could not identify any standard treatment guidelines, particularly for psychological treatments. |
| 21. Line 271, "mental health services organization," should instead be rewritten as "mental health police and regulation." | Thank you for this suggestion. However, we respectfully would want to maintain the current sub-heading as ‘mental health services organisation’. The data presented is mainly on how mental health services are organised, and the policy framework. |
| 22. Lines 273-282 tell the story of Ghana's mental health authorities. Mental health authorities and offices are not mental health organizations, and their main responsibilities might be recruiting mental health professionals, planning mental health services, coordinating, planning, and conducting supportive supervision, delivering training for health providers, and conducting monitoring and evaluation. Since the Mental Health Act is not part of the study results, it would be better if Ghana’s Mental Health Act 2012 (Act 846) were incorporated into the background of this section of the study. | Thank you for your comment. We would like to clarify that the data retrieved on the Mental Health Act is indeed part of the situation analysis findings. The PRIME tool has a section on ‘Mental health politics, policies and plans’.  The section on mental health services organisation would require data on the policy framework underpinning the organisation of mental health services in Ghana. |
| 23. Lines 284–297 described mental health services plans, authorities, responsibilities, and roles at the regional and district level. What about mental health care plans at each and every level of mental health facilities (i.e., at health posts, health centers, district hospitals, and tertiary hospitals)? Similarly, this situational analysis did not report results showing health facilities' quarterly and annual plans or performances regarding mental health services at health facilities. | Thank you for your comment. District mental healthcare plans are inclusive and would provide the blueprint for the organisation of mental health services in all health facilities, including community support systems.  Regarding your comment on quarterly and annual plans, our situation analysis reviewed annual health reports of each district covering 2019 and 2020, and medium-term development plans of the districts. As reported in our results, whilst there are no district mental healthcare plans, mental health services exist and are coordinated by the District Mental Health Focal Person (1 per district; usually a registered mental health nurse rank) with the assistance of sub-district coordinators. |
| 24. It is better if you summarize the two subtopics (availability of mental health services and human resources for mental health service provision) as follows: mental health professionals and mental health services at health facilities | Thank you for this suggestion. We have now renamed the sub-heading as ‘mental health professionals and mental health services at health facilities’ |
| 25. You stated that people with mild and moderate mental and neurological disorders got mental health services at community health centers and district hospitals. What about people with severe mental illnesses who met all admission criteria and require close monitoring? | Where mental health services are offered at ommunity health facilities, people with severe mental health conditions are referred to the district hospital. |
| 26. Line 384, table 3, description, needs revision. Please revise and rewrite it in a clear and brief manner. | We have now edited the table title to make it clearer. |
| 27. The topic of the first column of table 3 was not appropriately written; revise it as "mental health activities at mental health offices and health institutions." | Thank you for your suggestion. However, we prefer to maintain the current wording to ensure alignment with the domains in the PRIME situation analysis tool. |
| 28. In Table 3 column 1, topic 2, you state "personnel type." Rephrase it as "mental health professionals from various backgrounds." | Thank you for your suggestion. However, we prefer to maintain the current wording to ensure alignment with the domains in the PRIME situation analysis tool. |
| 29. According to this situational analysis, alcohol detoxification was possible without institutionalization in Tolon, Asunafo North, and Anloga districts. How is alcohol detoxification possible without admitting the clients to health facilities? What are the researcher’s justifications? | Thank you for this important observation. We have now checked with the key respondents from Bongo and they confirm that they’re able to briefly admit patients at the district hospital to provide the service. Anloga has no hospital and thus unable to provide this service. We have now made the correction. |
| 30. You have reported that severe mental health illness is treated at home with the support of families in cases from all study districts. But it is difficult to treat SMI at the home level. According to the mhGAP intervention guidelines, all individuals with SMI should be treated by a psychiatrist at a psychiatry clinic or unit.So, what are your justifications for this inappropriate treatment service for people with SMI? | Thank you for this important observation. This is what our situation analysis found. We agree the standard practice for managing SMI requires more advanced expertise especially at the acute stages. This unfortunately is not the practice on the ground as there are no psychiatrists in district hospitals. Mental health nurses are required to provide some support and this is done at home with the support of the family, normally through home-visits. |
| 31. What are the parameters of mental health service coverage? What are the incidence and prevalence of mental, neurological, and substance use conditions in your study districts? In order to measure or estimate mental health services and coverage at study sites, incidences and prevalence of mental health disorders must be stated.Your situational analysis did not show the actual number of people with MNS conditions who had gotten treatment at health facilities in study districts. Furthermore, these situational analyses fail to provide a clear and concise description of the treatment gaps in the study area. | Data on treatment coverage (proportion of people with MNS disorders in the districts accessing care) was challenging to assess because the parameters are not routinely assessed and reported. Given the importance of the treatment coverage indicator, we were only able to extrapolate using prevalence estimates for Ghana from the 2019 Global Burden of Disease study[1], the population of the district, and the total number of registered patients with mental health conditions for the top-3 mental health conditions.  The treatment gaps are many and these are summarised in table 3. We recorded a <1 percent treatment coverage and this is one of our key findings. |
| 32. Lines 364-366 require revision because they are unclear and the write-up is inadequate. | Thank you. This has now been rewritten. |
| 33. Line 374-375, table 4, 19 words, which is more than the standards, needs revision. | Thank you. We have now reduced the word count. |
| 34. Lines 384-385, able-5, 22 words, which exceeds the plos one journal standards | Thank you. We have now reduced the word count. |
| 35. Line 387, subtopic "Availability and sources of psychotropic medication." | We are not clear with this comment |
| 36. Similarly, the availability of a mental health plan, budget allocation, coordination, integration, and use of screening tools are not appropriately stated for each district's health offices. | Thank you for your comment. Most of this information is provided in Table 3. |
| 37. Revise the paragraph as "psychotropic medication for people with MNS conditions" and try to summarize and rewrite the availability and constant supply of essential psychotropic drugs by kinds (i.e., anti-psychotics, antihistamines, anti-depressants, and anxiolytics) at each and every level of health facilities. | Thank you for this suggestion. However, we respectfully would want to maintain the current sub-heading as ‘availability and sources of psychotropic medication’. We feel the data presented under this sub-section specifically addresses issues of availability and sources.  As indicated, the assessment clearly shows that availability and supply of psychotropic mediations is a challenge. All medications come through the district hospital and the mental health focal person does the allocation to all health facilities that provide mental health services. |
| 38. Table 6, last row and column 6, "Carbamazepine," is available at Asunafo North for epilepsy but not for mood stabilizers. Please, would you like to justify the reason behind these discrepancies? | Thank you for your comment. We have now double-checked this discrepancy and can confirm it was entered in error. Carbamazepine is available both as an anti-epileptic and mood stabilizer. |
| 39. Line 419, under subtopic "monitoring and evaluations," Did the district health offices and health facilities have clear plans to conduct monitoring and evaluation programs at each level of health facility? Did they have a well-established functional monitoring and evaluation team? | At the district level, the mental health focal person conducts supervision for colleague mental health professionals, but this is usually unstructured and constrained by budgetary challenges. |
| 40. This paragraph under the subtopic "monitoring and evaluation" is not clear and concise enough to be read and understood. So, please summarize and rewrite this paragraph in a clear and brief manner. | Thank you for your comment. We have now revised the section to make it clearer. |
| 41. Discussion: - lacks depth, is too shallow, and does not address other studies' findings from around the world. It is better to include "study limitations and strengths" as separate sub-headings. | Thank you for your comment. We have now included a sub-section on limitations and strengthens.  Ours is the only other reported situation analysis outside the PRIME studies. Our discussion thus was largely situated within the findings from the PRIME studies. |

I look forward to your reply in due course.

Sincerely,

Benedict Weobong

[1].	Global Burden of Disease Study 2019 (GBD 2019). Results [Internet]. Institute for Health Metrics and Evaluation (IHME). 2019 [cited November 2021]. Available from: <https://vizhub.healthdata.org/gbd-results/?params=gbd-api-2019-public/f3773ac8f33d1d1af22ebfd3dadd6770>.
