# Supplementary material for: Towards implementation of context-specific integrated district mental healthcare plans: A situation analysis of mental health services in five districts in Ghana
Source: PLoS One. 2023 May 18;18(5):e0285324. doi: 10.1371/journal.pone.0285324 (PMC10194950; doi:10.1371/journal.pone.0285324)
Supplement: Attachment — Submitted filename: Response to reviewers.docx [file pone.0285324.s003.docx]

Dear Dr Sharma,

Manuscript: PONE-D-22-23817, Towards implementation of context-specific integrated district mental health care plans: A situation analysis of mental health services in five districts in Ghana

Thank you for your comments regarding our further revised manuscript and the invitation to copy-edit. We have now completed a thorough copy-editing. All the revisions in the revised manuscript are in track changes.

I look forward to your reply in due course.

Sincerely,

Benedict Weobong

[1].	Global Burden of Disease Study 2019 (GBD 2019). Results [Internet]. Institute for Health Metrics and Evaluation (IHME). 2019 [cited November 2021]. Available from: <https://vizhub.healthdata.org/gbd-results/?params=gbd-api-2019-public/f3773ac8f33d1d1af22ebfd3dadd6770>.
