# Supplementary material for: Towards implementation of context-specific integrated district mental healthcare plans: A situation analysis of mental health services in five districts in Ghana
Source: PLoS One. 2023 May 18;18(5):e0285324. doi: 10.1371/journal.pone.0285324 (PMC10194950; doi:10.1371/journal.pone.0285324)
Supplement: Attachment — Submitted filename: COMMENTS & SUGGESTION for the 2nd times.docx [file pone.0285324.s004.docx]

PLOS ONE

**Manuscript Title**- Towards implementation of context-specific integrated district mental health care plans: A situation analysis of mental health services in five districts in Ghana.

**Manuscript Number**: PONE-D-22-23817

Dear authors! You are doing a great job. Thank you for conducting this study. The majority of my concerns regarding this manuscript were well addressed, and you made the manuscript acceptable for publication. However, I still have a few concerns and comments on the financial grant number, the treatment of severe mental illness at home by the patient's family, and the recommendation.

**Results**: - You have reported that severe mental health illness is treated at home with the support of families in cases from all study districts. I don't know the Ghanaian context, but it is difficult to treat SMI at the home level. According to the mhGAP intervention guidelines, all individuals with SMI should be treated by a psychiatrist at a psychiatry clinic or unit. So, what are your justifications for this inappropriate treatment service for people with SMI? Do you believe that severe mental illness can be treated at home with the support of family, if appropriate? Since some patients with severe mental illness (e.g., those with schizophrenia, bipolar I with psychotic features, or MDD with psychotic features) might be present with violent behavior, agitation, and suicidal intentions that need immediate management by mental health professionals by using different psychotropic drugs and seclusion.

The financial disclosure section has no grant number.

Write recommendations based on your study findings and insert a separated subtopic as "Recommendations."
