# Supplementary material for: Towards implementation of context-specific integrated district mental healthcare plans: A situation analysis of mental health services in five districts in Ghana
Source: PLoS One. 2023 May 18;18(5):e0285324. doi: 10.1371/journal.pone.0285324 (PMC10194950; doi:10.1371/journal.pone.0285324)
Supplement: Attachment — Submitted filename: Response to reviewers.docx [file pone.0285324.s005.docx]

Dear Dr Sharma,

Manuscript: PONE-D-22-23817, Towards implementation of context-specific integrated district mental health care plans: A situation analysis of mental health services in five districts in Ghana

Thank you for your comments regarding our further revised manuscript and the invitation to revise.

Kindly refer to the response below:

**Query 1**

**Results**: - You have reported that severe mental health illness is treated at home with the support of families in cases from all study districts. I don't know the Ghanaian context, but it is difficult to treat SMI at the home level. According to the mhGAP intervention guidelines, all individuals with SMI should be treated by a psychiatrist at a psychiatry clinic or unit. So, what are your justifications for this inappropriate treatment service for people with SMI? Do you believe that severe mental illness can be treated at home with the support of family, if appropriate? Since some patients with severe mental illness (e.g., those with schizophrenia, bipolar I with psychotic features, or MDD with psychotic features) might be present with violent behavior, agitation, and suicidal intentions that need immediate management by mental health professionals by using different psychotropic drugs and seclusion.

**Response:** Respectfully, we want to maintain our previous response to this question. As we indicated, this was a situation analysis of mental health services in the selected five districts. What we report here is what we found as current practices, and these are not our recommendations; the design of the study is to describe and characterise the current state of mental health services. We agree the standard practice for managing SMI requires more advanced expertise especially at the acute stages. This unfortunately is not the practice on the ground as there are no psychiatrists in district hospitals. Mental health nurses are required to provide some support, and they provide this care at home with the support of the family, normally through home-visits. We have now shared our thoughts on the implications of this finding in the discussion (lines 491 to 496).

**Query 2**

The financial disclosure section has no grant number.

**Response:** We have now inserted the grant number: PO8604

**Query 3**

Write recommendations based on your study findings and insert a separated subtopic as “Recommendations.”

**Response:** The nature of the study and our approach to the discussion meant that we made recommendations as we discussed key implications of the findings. For example, in line 486 to 487, we provided specific recommendations regarding our findings on the state of mental health information systems (under implications for health systems for mental health). Lines 523 to 525 and 542 to 546 has recommendations on how to deal with data quality/completeness issues. Lines 539 to 540 contains recommendations regarding how to achieve integrated mental healthcare by leveraging existing guidelines. Similarly, in lines 548 to 551 we made recommendations regarding community reintegration and rehabilitation of people with mental illness. With this approach, we are concerned having a separate section on recommendations will duplicate the text, and we prefer to not make these changes. If having a separate subheading for recommendations is a strict journal requirement, we would be happy to re-arrange the text, to delete the earlier embedded recommendations, and have a separate section for recommendations.

I look forward to your reply in due course.

Sincerely,

Benedict Weobong

[1].	Global Burden of Disease Study 2019 (GBD 2019). Results [Internet]. Institute for Health Metrics and Evaluation (IHME). 2019 [cited November 2021]. Available from: <https://vizhub.healthdata.org/gbd-results/?params=gbd-api-2019-public/f3773ac8f33d1d1af22ebfd3dadd6770>.
